# Young People With and Without Intellectual Disability Accessing Mental Health Services: Evaluating Psychosocial Functioning Outcomes Using Electronic Health Records

**DOI:** 10.1111/jir.70066

**Published:** 2025-11-29

**Authors:** Z. Sienna Pan, Carlo Schuengel, Barry Coughlan, Lianne Bakkum, Matt Woolgar, Sarah Carman, Robbie Duschinsky

**Affiliations:** ^1^ Department of Public Health and Primary Care University of Cambridge Cambridge UK; ^2^ Department of Educational and Family Sciences Vrije Universiteit Amsterdam Amsterdam The Netherlands; ^3^ Department of Psychology National College of Ireland Dublin Ireland; ^4^ Amsterdam Public Health research institute The Netherlands; ^5^ Institute of Psychiatry, Psychology and Neuroscience King's College London London UK; ^6^ South London and Maudsley NHS Foundation Trust London UK

**Keywords:** child psychiatry, epidemiology, intellectual disability, mental health, neurodevelopmental condition, outcome

## Abstract

**Background:**

There is growing recognition that many young people (<18 years) with intellectual disability (ID) may benefit from psychosocial support provided by mental health services, yet intervention outcomes have not been robustly evaluated.

**Method:**

Data from 1986 episodes of care for young people with ID and 3968 matched episodes for those without ID were extracted from electronic health records of the South London and Maudsley NHS Foundation Trust (2001–2023). Psychosocial functioning was assessed using the Children's Global Assessment Scale (CGAS).

**Results:**

ID frequently co‐occurred with other neurodevelopmental conditions and behavioural difficulties (prevalence > 50%). CGAS scores at service entry positively predicted CGAS scores at discharge; however, this association weakened in the presence of ID and co‐occurring pervasive developmental disorders or hyperkinetic disorders. ID was associated with lower CGAS scores at discharge than those without ID. Within the ID group, young people with severe/profound ID and comorbidities demonstrated greater rates of improvement than those with severe/profound ID only. 20% of young people with ID showed clinically significant improvement at discharge (reliable change index ≥ 1.96). Despite this improvement, 80% of the same group continued to experience substantial impairment (CGAS < 61).

**Conclusions:**

Most young people with ID remained substantially impaired at discharge, highlighting the complexity of their needs and the importance of sustained, targeted support. Further research should examine specific intervention types and treatment trajectories in this population.

**Abbreviations**
CAMHS: Child and Adolescent Mental Health Services
CD: conduct disorder
CGAS: Children's Global Assessment Scale
CI: confidence interval
CRIS: Clinical Record Interactive Search
HD: hyperkinetic disorders
ICD‐10: International Classification of Disease: 10th Edition
ID: intellectual disability
IMD: index of multiple deprivation
NHS: National Health Services
NICE: National Institute for Health and Care Excellence
PDD: pervasive developmental disorders
SD: standard deviation
SLaM: South London and Maudsley NHS Mental Health Trust

## 1 Introduction

Intellectual disability (ID) is estimated to be present in 1%–3% of the general population globally, including approximately 300 000 children in England (National Institute for Health and Care Excellence (NICE) 2021; D. R. Patel et al. 2020). ID is clinically characterised by neurodevelopmental differences in cognitive and adaptive functioning that emerge during early developmental periods (American Psychiatric Association 2013; World Health Organisation 2022), with impairments in conceptual, social, and practical skills persisting into adulthood (Cluley 2018; D. Patel et al. 2018).

ID frequently co‐occurs with other neurodevelopmental conditions, behavioral problems, and mental health difficulties, particularly autism spectrum disorders, attention deficit hyperkinetic disorders, conduct disorders (CDs), and anxiety disorders (Buckley et al. 2020; Emerson et al. 2023; Totsika et al. 2022; van der Mheen et al. 2024). However, these co‐occurring conditions can be obscured by the presence of ID, leading to missed or delayed diagnoses of potentially treatable conditions and creating barriers to timely, appropriate support (Whittle et al. 2018; Mason and Scior 2004).

In England, Child and Adolescent Mental Health Services (CAMHS) have the statutory responsibility to support young people with a wide range of mental health needs, including low mood, significant life changes, and psychiatric disorders. In this study, the term *intervention* refers to an episode of care provided by CAMHS, which may include assessment, care planning and/or treatment. Depending on complexity, some young people with ID and mental health difficulties may be referred to specialist or highly specialist services for targeted and intensive support. However, it remains unclear how effectively CAMHS meets their needs, and what factors may contribute to intervention outcomes.

Concerns about determining the effectiveness of interventions for this population have persisted for decades, given the methodological limitations of previous studies and the challenges of conducting robust service‐level research. Systematic reviews consistently report that most studies in this area are of low quality, often constrained by small sample sizes, suboptimal study designs, and lack of comparison groups (Byrne 2022; Vereenooghe et al. 2018; Vereenooghe and Langdon 2013). Although some longitudinal studies have highlighted that individuals with more severe ID tend to have poorer clinical outcomes (De Ruiter et al. 2008; Einfeld et al. 2006), most studies have been limited to individuals with mild or moderate ID, underrepresenting those with severe or profound disability (Tapp et al. 2023; Vereenooghe et al. 2018; Vereenooghe and Langdon 2013).

To address these research gaps, this study aims to evaluate the psychosocial functioning outcomes in a large sample of young people with and without ID by addressing the following research questions:

1. What is the association between ID diagnosis and psychosocial functioning at service entry and discharge?
2. What is the association between ID severity and psychosocial functioning improvement at discharge?
3. Does the association between ID and psychosocial functioning at discharge differ by co‐occurring psychiatric conditions?
4. What are the clinical outcomes of young people with and without ID?

## 2 Methods

### 2.1 Participants and Procedures

Patient data were extracted from the Clinical Record Interactive Search (CRIS) database, which holds deidentified electronic health records from South London and Maudsley NHS Mental Health Trust (SLaM) (Perera et al. 2016; Stewart et al. 2009). SLaM provides mental health services for over 1.3 million residents in the London boroughs of Croydon, Lambeth, Lewisham, and Southwark.

The integrated electronic health record system has been comprehensively used across all services in SLaM since 2006, followed by the establishment of CRIS in 2008 (Stewart et al. 2009). Patient information from 1999 onwards was migrated from previous independent systems at the time of implementation. Most diagnoses of ID (98.18%, *N* = 2969) were entered into structured fields after 01 January 2007. The case group in this study included all inpatient and outpatient episodes of care for young persons who:

1. Have ever received an ICD‐10 structured diagnosis of ID (F70–F79) before age 18, recorded by 22 April 2023 (end of study window);
2. Have been discharged from the episode of care;
3. Have had two recorded CGAS scores between referral accepted date and discharge date in the same episode. The first CGAS score is the earliest record in the rating period of ‘initial assessment,’ ‘inpatient admission,’ ‘other review’ or ‘care programme approach (CPA) review.’ The last CGAS score is the latest record in the rating period of ‘discharge from Trust,’ ‘inpatient discharge’ or ‘other transfer within Trust’.

An episode of care is a period of care provided to a patient to treat a clinical condition, beginning at service entry and ending at discharge from a CAMHS team. For young people with multiple episodes, each episode was analysed separately, as it could be provided by a different team within SLaM.

By April 2023, 3024 young people had a structured, primary or secondary diagnosis of ID in one of their referrals. Of these, 1986 episodes of care for 1636 young people across 72 CAMHS service teams had completed CGAS assessments at service entry and discharge. The earliest structured diagnosis of ID was recorded on 25 July 2002.

Each ID case was matched with two non‐ID controls based on baseline covariates, using propensity scores to balance systematic differences (Austin 2011a; Zhao et al. 2021). The control group was randomly selected from 20 000 episodes of care of young people diagnosed with mental, behavioural or neurodevelopmental disorders (F00–F99), excluding ID (F70–79), within the same study window. Controls also met the inclusion criteria (2) and (3) above.

**FIGURE 1:**
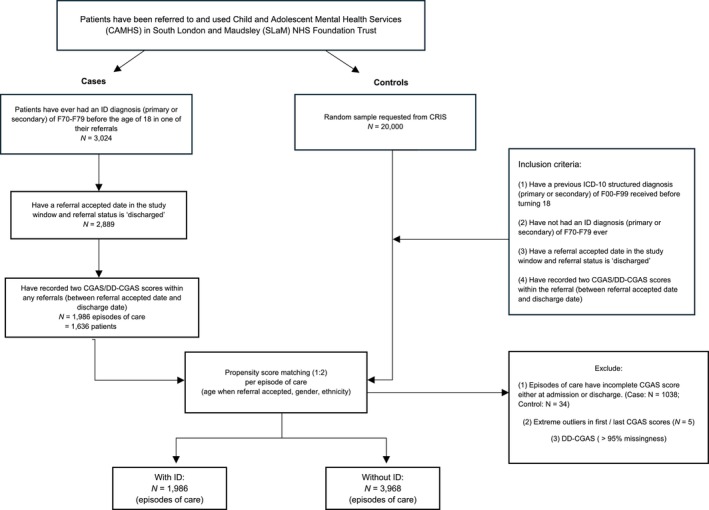
Flow chart of participants in the case–control study.

A total of 3968 control episodes, across 78 CAMHS service teams, were successfully matched with 1986 ID episodes. A participant flow chart is presented in Figure 1.

ID severity was categorised within the records as mild (F70), moderate (F71), severe (F72), profound (F73) and other/unspecified (F7879). Because other ID and unspecified ID do not necessarily indicate severity, they were recoded into one group.

Psychiatric comorbidities were investigated using the broader level of ICD‐10 diagnostic codes. For example, F84.0–F84.9 were coded into F84. Considering the sample size and study power, only the three most prevalent diagnoses in the ID group were included in the statistical models: F84 pervasive developmental disorders (PDDs), F90 hyperkinetic disorders (HDs) and F91 CDs. Diagnostic coding in CRIS follows the ICD‐10 multiaxial classification system (WHO 1996), in which ID (F70–F79) falls under Axis III: Intellectual Level, whereas other neurodevelopmental and behavioural disorders fall under Axis I: Clinical Psychiatric Syndromes.

In this study, the term *comorbidity* refers to a comorbid diagnosis of at least one psychiatric condition, which falls under the ICD‐10 Axis I: Clinical Psychiatric Syndromes, during an episode of care for young people with ID.

#### 2.1.1 Ethics Statement

CRIS was granted approval by the South Central–Oxford C Research Ethics Committee for secondary analyses of anonymised data (reference 23/SC/0257). Approval to conduct the present study was granted by the CRIS Oversight Committee (22‐085).

#### 2.1.2 Representativeness of the CRIS cohort

The index of multiple deprivation (IMD) of the CRIS cohort was compared with the national IMD data in England to test for the sample representativeness using two‐sample *t* tests. IMD data from 2010 were used, as this year had the highest frequency of diagnoses in the CRIS cohort (*N* = 2519), and 2012 was the median year of diagnosis.

### 2.2 Measure of Psychosocial Functioning

The overall psychosocial functioning of young persons was measured by the *Children's Global Assessment Scale* (CGAS; Shaffer et al. 1983), which assesses the following four domains of functioning: self‐care, communication, social behaviour and school/academic performance. A single score of global functioning is yielded, ranging from 1 (most impaired) to 100 (superior functioning). The scale is divided into 10‐point intervals that represent different degrees of functioning. CGAS is the most widely used tool for measuring global functioning in SLaM and is considered sufficiently reliable for clinical use (Bird, Canino, et al. 1987; Lundh et al. 2010; Shaffer et al. 1983).

The Developmental Disabilities modification of the Children's Global Assessment Scale (DD‐CGAS; Wagner et al. 2007) data was also extracted, as illustrated in Figure 1. However, because of over 95% missing data, only the CGAS was used.

### 2.3 Missing Data

A small proportion of data was missing for ethnicity (5.7%) and IMD (1.3%). Missing data were handled using multiple imputation with the *‘mice’* package (v3.18.0; van Buuren and Groothuis‐Oudshoorn 2011). Seven variables were identified as predictors of missingness and were included in the imputation model. Little's tests (Li 2013; Little 1988) suggested that missingness was not completely at random and was dependent on the identified covariates. Five imputed datasets were generated. Diagnostic plots showed that the observed data and imputed data converged well (Supplementary Material Tables S1–S2; Figure S1).

### 2.4 Statistical Analysis

The propensity score was estimated with a logistic regression predicting the diagnosis of ID based on the matching covariates. Covariates associated with the exposure (ID diagnosis) and the outcome (first CGAS score), or with the outcome alone, were included in the matching model (Zhao et al. 2021). To optimise matching, nearest neighbour calliper matching without replacement was used (Austin 2010, 2011b, 2014; Lunt 2014). A non‐ID control with a propensity score (in random order) nearest to an ID case, within a calliper width of 0.2 of the standard deviation of the logit of the propensity score, was selected. Matching was performed using the R package ‘*MatchThem*’ (v1.2.1; Pishgar et al. 2021) after multiple imputation.

**FIGURE 2:**
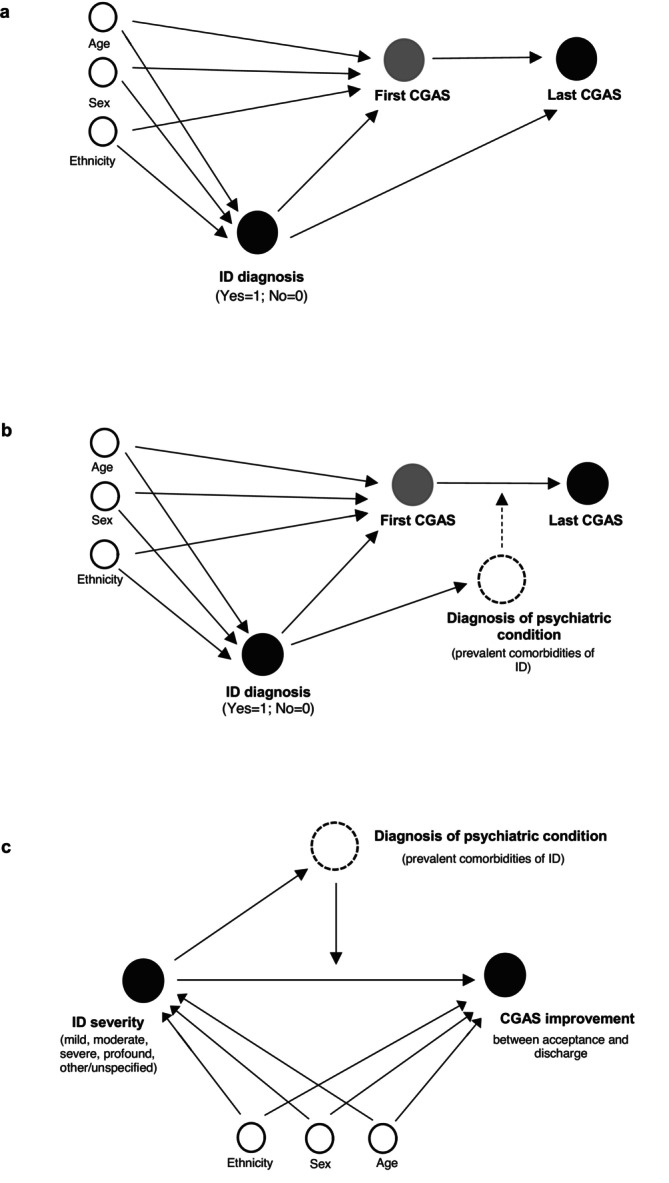
Directed acyclic graphs of the present study. (a) Model 1: mediation analysis. (b) Model 2: three‐way interaction analysis. (c) Model 3: two‐way interaction analysis (within the ID group).

Directed acyclic graphs of the statistical analyses are shown in Figure 2. For Models 1–3, which correspond to Figure 2a–c, multilevel models were implemented to account for CAMHS service team clustering. To assess the degree of clustering, unconditional means models with random intercepts for CAMHS service teams were first estimated.

We examined two outcomes: (1) CGAS scores at discharge and (2) CGAS improvement (discharge minus service entry). Each model first tested the direct association between independent and dependent variables and then added mediators or moderators based on study design. The multilevel mediation analysis was conducted using the ‘*lavaan.mi*’ package (v0.1.0; Jorgensen 2025). All other multilevel models were estimated using the ‘*lme4*’ package (v1.1.37; Bates et al. 2015). Estimates were pooled across imputations using Rubin's rules (Rubin 1987), as implemented in the ‘*mice*’ and ‘*lavaan.mi*’ packages.

Severe and profound IDs were treated as distinct groups in the direct association analysis with psychosocial functioning outcomes. However, they were combined into a single category for interaction analyses of ID and comorbidity, as the sample sizes of young people with ‘severe ID + comorbidity’ and ‘profound ID + comorbidity’ were too small to perform reliable estimation.

All analyses were conducted in R (Version 4.4.0) in the RStudio software environment (Version 2024.4.1.748) (Posit Team 2024; R Core language Team 2024). Statistical significance level was set at 0.05.

### 2.5 Clinical Significance Analysis

#### 2.5.1 Individual‐Level Change

Individual persons' change between the start and the end of the episode of care was evaluated using Jacobson and Truax's approach (1991).

A reliable change index ≥ 1.96 indicates that it is unlikely that the change in CGAS score is reflecting a real change. A cutoff score of 60 on the CGAS is commonly used as a standard for definite pathology (Bird, Yager, et al. 1990; Roest et al. 2021). Scores below 61 range from ‘variable functioning with sporadic difficulties’ (51–60) to ‘needs constant supervision’ (1–10), while scores above 60 indicate ‘some difficulty in a single area, but generally functioning pretty well’ (61–70) to ‘superior functioning’ (91–100).

Thus, individuals' changes after interventions were categorised as follows: (1) ‘Improved + recovered’: reliable change index ≥ 1.96 and last CGAS score > 60; (2) ‘Improved’: reliable change index ≥ 1.96; (3) ‘Recovered’: last CGAS score > 60; (4) ‘Unchanged’: last CGAS score is the same as the first CGAS score; (5) ‘Worsened’: reliable change index ≤ −1.96.

#### 2.5.2 Mean‐Level Change

Paired *t* tests were conducted to examine mean differences in CGAS scores before and after interventions. Effect sizes were calculated using Cohen's *d*.

All clinical significance analyses used the original sample without multiple imputation or propensity score matching.

## 3 Results

### 3.1 Summary Statistics

As shown in Table 1, 68% of young people with ID were male, and around half of the sample identified as an ethnic minority. The mean first CGAS score in the case group was 41.5, which was 11.3 points lower than that of the control group. The mean IMD of the CRIS cohort was significantly higher (more deprived) than the national average IMD of England (CRIS: 27.8 vs. England: 19.2; *p* < 0.001). Compared with the control group, young people with ID had longer durations of service engagement per episode (mean: 97.2 weeks vs. 65.3 weeks; *p* < 0.001).

**TABLE 1:** Descriptive table of the case and control groups before propensity score matching.

Variable		Case	Control	*p*	Missing (%)
Observations (per episode)	*n* (%)	1986 (9.0)	19 966 (91.0)	<0.001	1539 (7.0)
Age when referral accepted	Mean (SD)	11.2 (3.7)	12.2 (3.7)	<0.001	0 (0)
Age at earliest diagnosis	Mean (SD)	11.7 (3.6)	12.1 (3.7)	<0.001	0 (0)
Ethnicity				<0.001	1260 (5.7)
White	*n* (%)	941 (47.4)	10 452 (52.3)		
Asian	*n* (%)	142 (7.2)	868 (4.3)		
Black	*n* (%)	602 (30.3)	4777 (23.9)		
Mixed	*n* (%)	143 (7.2)	2040 (10.2)		
Other	*n* (%)	66 (3.3)	661 (3.3)		
Gender					2 (<0.01)
Female	*n* (%)	628 (31.6)	10 146 (50.8)		
Male	*n* (%)	1358 (68.4)	9722 (48.7)		
Not specified	*n* (%)	0 (0)	12 (0.1)		
Other	*n* (%)	0 (0)	84 (0.4)		
IMD	Mean (SD)	28.2 (12.4)	27.7 (11.8)	0.052	277 (1.3)
First CGAS score	Mean (SD)	41.5 (14.6)	52.8 (11.7)	<0.001	0 (0)
Last CGAS score	Mean (SD)	47.9 (15.9)	63.0 (13.1)	<0.001	0 (0)
Duration of service engagement (weeks per episode)	Mean (SD)	97.2 (120.7)	65.3 (82.3)	<0.001	183 (0.83)
	Minimum	0.43	0.14		
	Maximum	888.9	821.9		
	Median	52.1	38.6		

Prevalent comorbidity of ID		Case	Control
F84 Pervasive developmental disorders	*n* (%)	1083 (54.5)	2404 (12.0)
F90 Hyperkinetic disorders	*n* (%)	462 (23.3)	1979 (9.9)
F91 Conduct disorders	*n* (%)	155 (7.8)	1115 (5.6)

Unconditional means models with random intercepts for CAMHS service team indicated intraclass correlation coefficients (ICCs) of 0.179 for CGAS scores at discharge and 0.165 for CGAS improvement, suggesting that 17.9% and 16.5% of the variance in these outcomes, respectively, was attributable to clustering by team. Given this evidence, all subsequent models included a random intercept for service team.

Without considering mediators or moderators, a higher CGAS score at discharge was significantly associated with not having an ID (*β*_ID_ = −5.17, 95% CI [−5.87; −4.47]), having a higher baseline CGAS score (*β*_1cgas_ = 0.68, 95% CI [0.66; 0.70]), being older at service entry (*β*_age_ = 0.25, 95% CI [0.14; 0.31]), being female (*β*_male_ = −1.19, 95% CI [−1.85; −0.53]) and having a longer duration of service engagement (*β*_duration_ = 0.007, 95% CI [0.004; 0.009]; Supplementary Material Table S5).

### 3.2 Model 1: Mediation Role of Psychosocial Functioning at Service Entry

As shown in Table 2, the total indirect effect yielded an estimate of −6.31 (95% CI [−7.44; −5.22]). The direct effect (controlled for the mediator) was smaller than the total effect (|−5.30| < |−11.61|), indicating that the first CGAS score mediated part of the relationship between ID diagnosis and last CGAS score. The majority of the total effect was mediated through the first CGAS, but there remained a direct negative effect of ID diagnosis on the last CGAS that was not fully explained by the first CGAS score.

**TABLE 2:** Mediation analysis for the case and control groups. Missing data were handled using multiple imputation.

	*β*	95% CI
Direct effect^a^	−5.30	[−6.50; −4.05]
Total indirect effect^b^	−6.31	[−7.44; −5.22]
Total effect^c^	−11.61	[−13.04; −10.15]

^a^ ID case → last CGAS score (adjusting for first CGAS score).

^b^ ID case → first CGAS score → Last CGAS score.

^c^ ID case → last CGAS score.

### 3.3 Model 2: Moderation Role of ID and Comorbidities

The results of the two‐way interaction analysis indicated that the positive effect of the first CGAS score on the last CGAS score was stronger for young people with ID than for those without (*β*_1cgas:id_ = 0.24, *p* < 0.001, 95% CI [0.19; 0.29]). When controlling for the first CGAS score, young people with an ID diagnosis were associated with a decrease of −16.66 units in the last CGAS score, compared to those without ID (*p* < 0.001, 95% CI [−19.05; −14.27]) (Supplementary Material S5).

The three‐way interaction analyses, however, found that the positive two‐way interaction effect of the first CGAS score and ID diagnosis on the last CGAS score was weakened in the presence of PDD (*β*_1cgas:id:pdd_ = −0.22, *p* < 0.001, 95% CI [−0.33; −0.11]) and HD (*β*_1cgas:id:hd_ = −0.20, *p* < 0.001, 95% CI [−0.32; −0.07]).

**FIGURE 3:**
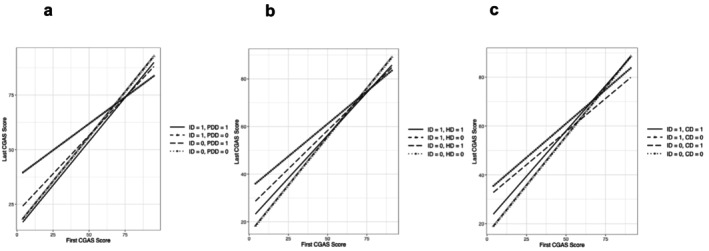
Visualisation of three‐way interaction models for three comorbidity groups: (a) ID × PDD; (b) ID × HD; (c) ID × CD. Note for abbreviations: ID: intellectual disability; PDD: pervasive developmental disorders; HD: hyperkinetic disorders; CD: conduct disorder.

As illustrated in Figure 3, the slopes of the comorbidity groups (‘ID + PDD/HD/CD’) were slightly flatter than those of the ID‐only groups. While individuals without ID and comorbidity had the highest starting point, the flatter slope suggests that their improvement after interventions was less pronounced than that of the other three groups.

### 3.4 Model 3: Within‐Group Analysis

**FIGURE 4:**
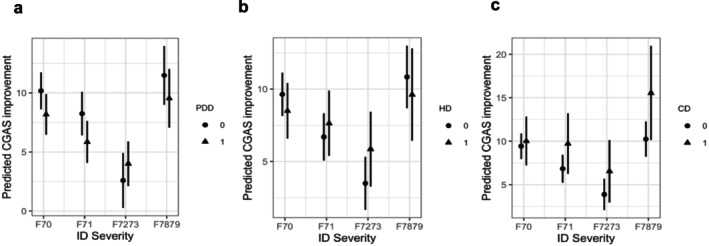
Visualisation of two‐way interaction models for three comorbidity groups: (a) ID × PDD; (b) ID × HD; (c) ID × CD. Note for abbreviations: ID: intellectual disability; PDD: pervasive developmental disorders; HD: hyperkinetic disorders; CD: conduct disorders.

Interaction analyses found that the negative association between severe/profound ID and CGAS improvement was significantly weakened in the presence of PDD and HD (*β*_F7273:pdd_ = 3.42, *p* < 0.05, 95% CI [0.86; 5.97]; *β*_F7273:hd_ = 3.50, *p* < 0.01, 95% CI [0.63; 6.36]) (Supplementary Material S6). The interaction plots (Figure 4) also illustrate that young people with severe/profound ID and co‐occurring PDD or HD had significantly higher predicted improvements in psychosocial functioning than those with severe/profound ID only.

A similar pattern was observed in the descriptive table of group‐level changes (Supplementary Material Table S7). Although young people with severe/profound ID and comorbidity generally had lower baseline CGAS scores than those in the mild and moderate ID and comorbidity groups, their mean improvement from service entry to discharge was the highest across all comorbidity conditions, except for the other/unspecified ID group. Additionally, young people with severe/profound ID and comorbidity had greater mean improvement in CGAS than those with severe/profound ID only (Δ CGAS_F7273_ = 5.89 vs. Δ CGAS_F7273 + PDD_ = 6.55; Δ CGAS_F7273 + HD_ = 9.33; Δ CGAS_F7273 + CD_ = 9.57).

The results of complete case analysis and multiple imputation for Model 1–3 were comparable (Supplementary Material Tables S4–S6).

### 3.5 Clinical Significance Analysis

Both the case and control groups showed significant improvement in psychosocial functioning between service entry and discharge (Table 3a). The control group started with better functioning and demonstrated greater clinical improvement based on mean CGAS scores and the percentage of young people moving out of the clinical range (CGAS score > 60), compared to the case group (Table 3b).

**TABLE 3:** Results of clinical significance analyses.

(a) Preintervention and postintervention means on the CGAS divided into case (at different severity levels) and control groups.						
	*n*	First CGAS	Last CGAS	Paired *t* test	Effect size	Δ CGAS score
		Mean (SD)	Mean (SD)	*p*	Cohen's *d* (CI)	Mean (SD)
**Case**	1986	41.5 (14.6)	47.9 (15.9)	*p* < 0.001	0.43 [0.36; 0.49]	6.48 (10.6)
Mild ID	800	47.7 (11.6)	54.6 (12.2)	*p* < 0.001	0.58 [0.48; 0.68]	6.9 (10.9)
Moderate ID	558	42.5 (12.6)	47.9 (14.2)	*p* < 0.001	0.41 [0.29; 0.52]	5.44 (10.0)
Severe ID	393	30.1 (13.5)	36.4 (15.6)	*p* < 0.001	0.43 [0.29; 0.57]	6.32 (10.2)
Profound ID	54	17.6 (13.4)	21.4 (15.1)	*p* = 0.006	0.27 [−0.12; 0.65]	3.80 (9.92)
Other/unspecified ID	181	42.7 (13.2)	51.6 (13.8)	*p* < 0.001	0.66 [0.45; 0.87]	8.93 (11.6)
**Control**	19 966	52.8 (11.7)	63.0 (13.1)	*p* < 0.001	0.82 [0.80; 0.84]	10.2 (12.8)

(b) Frequency and percentage of clinically significant change in the case and control groups.									
Measure: CGAS	*n*	Improved + recovered	Improved	Recovered	Unchanged	Worsened	First CGAS	Last CGAS	% Moved out of clinical range
							< 61	< 61	
		*n* (%)	*n* (%)	*n* (%)	*n* (%)	*n* (%)	*n* (%)	*n* (%)	
**Case**	1986	197 (9.9)	393 (19.8)	399 (20.1)	606 (30.5)	35 (1.8)	1837 (92.5)	1587 (79.9)	12.6
**Control**	19 966	5413 (27.1)	6516 (32.6)	10 929 (54.7)	4006 (20.1)	287 (1.4)	15 301 (76.6)	9037 (45.3)	31.4

(c) Frequency and percentage of clinically significant change among young people with different severities of intellectual disability.									
Measure: CGAS		Improved + recovered	Improved	Recovered	Unchanged	Worsened	First CGAS	Last CGAS	% Moved out of clinical range
							< 61	< 61	
ID severity	*n*	*n* (%)	*n* (%)	*n* (%)	*n* (%)	*n* (%)	*n* (%)	*n* (%)	
Mild	800	89 (11.1)	140 (17.5)	234 (29.2)	242 (30.2)	<9 (<1)	711 (88.9)	566 (70.8)	18.1
Moderate	558	38 (6.8)	70 (12.5)	90 (16.1)	179 (32.1)	<9 (<5)	518 (92.8)	468 (83.9)	9.0
Severe	393	19 (4.8)	62 (15.8)	29 (7.4)	124 (31.6)	<9 (<5)	388 (98.7)	364 (92.6)	6.1
Profound	54	0 (0)	<9 (<10)	<9 (<5)	20 (37.0)	<9 (<5)	53 (98.1)	53 (98.1)	0
Other/unspecified	181	23 (12.7)	45 (24.9)	45 (24.9)	41 (22.7)	<9 (<1)	167 (92.3)	136 (75.1)	17.1

*Note:*

(1) Values less than 9 and percentages less than 5% were reported as a range.

(2) Improved and recovered: reliable change index ≥ 1.96 + last CGAS > 60, Improved: reliable change index ≥ 1.96, recovered: last CGAS > 60, unchanged: first CGAS score = last CGAS score, worsened: reliable change index ≤ −1.96.

(3) ICD‐10 coding system: F70 = mild ID, F71 = moderate ID, F72 = severe ID, F73 = profound ID, F78 = other ID, F79 = unspecified ID.

Among young people with ID, 10% showed reliable ‘improvement + recovery’ after interventions, while 31% showed no change in psychosocial functioning, and only 2% had a worsened condition. Additionally, 683 episodes of care (34%) showed an increase in functioning level (last CGAS>first CGAS), but the improvement was not strong enough to meet the defined categories for clinically reliable change.

The proportion of young people with ID scoring in the clinical range decreased from 93% at service entry to 80% at discharge, although a significant proportion of them still experienced clinically significant impairment after interventions. In contrast, the control group showed better outcomes, with 31% of young people without ID moving out of the clinical range and 45% remaining.

Among the 55 young people with profound ID, none achieved clinical ‘improvement + recovery,’ while 37% experienced unchanged levels of psychosocial functioning impairment (Table 3c).

Overall, without considering comorbidities, young people with less severe forms of ID tended to have better outcomes in terms of clinical ‘improvement’ and ‘recovery’ compared to those with more severe forms. Although young people with severe/profound ID and a prevalent comorbidity, on average, showed the greatest magnitude of CGAS improvement compared to the mild and moderate ID with comorbidity groups, their functioning level at service entry was the lowest. Moreover, over 90% of them still experienced clinical impairments at discharge (Supplementary Material Table S7).

## 4 Discussion

### 4.1 ID Diagnosis and Psychosocial Functioning Outcome

The present study evaluated factors associated with psychosocial functioning outcomes of CAMHS interventions for young people with ID compared to a matched control group. Both groups showed improvements at discharge, but the improvements were generally smaller among those with ID. This difference was partly explained by lower psychosocial functioning at service entry among the ID group, suggesting that the starting point plays a key role in intervention outcomes. Poor psychosocial functioning at service entry may also reflect the mental health needs of the ID population. While ID itself is not typically a reason for mental health service referral, our study, consistent with findings from another study at SLaM (Coughlan et al. 2025), highlights the high prevalence of neurodevelopmental conditions and behavioural difficulties in mental health settings. Our findings suggest that co‐occurring psychiatric presentations or impairments in psychosocial functioning among young people with ID, which could include difficulties in emotional regulation, social interaction, or adaptive functioning, may contribute to distress and necessitate mental health support. Clinically, these findings highlight the importance of early detection of symptoms and timely intervention to prevent further functional deterioration.

### 4.2 ID Severity, Comorbidities, and Psychosocial Functioning Outcome

Co‐occurring psychiatric conditions were present in more than half of young people with ID. Within the ID group, young people with severe/profound ID and PDD or HD showed greater predicted improvement in psychosocial functioning, compared to those with severe/profound ID only. Furthermore, the mean functional improvements were highest among young people with severe/profound ID and comorbidities, compared to those with mild or moderate ID and comorbidities. These differences within the ID group raise questions about the factors linked to treatment trajectories for young people with varying levels of severity and complexity.

Previous research found that people with severe and complex conditions are likely to receive combined psychological and pharmacological treatments, or specialised support, which positively contributes to treatment outcomes (Embracing Complexity 2019; Reale et al. 2017). As shown in Reale's study of an Italian cohort (2017), young people with comorbid ADHD and learning disorders reported higher rates of receiving treatments and showed greater improvement after intervention compared to young people with ADHD only. However, as the authors highlighted, access to treatments is often constrained by systemic issues like waiting times and clinical prioritisation, which are also pressing concerns in the UK's NHS (Edbrooke‐Childs and Deighton 2020; Punton et al. 2022). Longer waiting times are linked to lower engagement and higher drop‐out rates in subsequent treatments, which are associated with poorer outcomes (Kreyenbuhl et al. 2009; Reichert and Jacobs 2018; Westin et al. 2014). These findings highlight the need for future studies examining waiting times, treatment trajectories, and outcomes among young people with different levels of ID severity and complexity.

### 4.3 Clinical Outcomes and Service Engagement

Clinical significance analysis showed that, despite improvements, 80% of young people with ID continued to experience clinical symptoms of psychosocial functioning impairment at discharge (CGAS < 61). Young people with less severe ID tended to have better outcomes in terms of clinical ‘improvement’ and ‘recovery.’ While the scores for young people with severe ID and comorbidities reflected very significant impairment at service entry, and many still experienced clinical impairments at discharge, there were nonetheless noticeable improvements.

The duration of service engagement varied widely in both case and control groups. Some young people remained with the trust for prolonged periods before discharge, but some had multiple, shorter episodes of care. In the latter scenario, the total time span from a child's first episode to their fifth could exceed 5 years. Subsequent episodes did not necessarily reflect re‐referral but could also represent referral to a different team within the trust. These findings highlight the heterogeneity in service use and potential challenges with continuity of care and engagement across CAMHS teams.

### 4.4 Strengths and Limitations

Our study adopted a propensity score matching approach to minimise systematic bias by matching young people with ID to those without ID based on sociodemographic characteristics and service engagement durations. Using routinely collected clinical data, we examined CAMHS intervention outcomes across all levels of ID severity while accounting for prevalent comorbidities. Through robust statistical analyses, including multilevel models with a random intercept for service team and clinical significance testing, the study provides comparative evidence on psychosocial functioning outcomes for young people with and without ID. These findings have important implications for future research and clinical service planning.

As the CGAS provides a holistic measure of functioning, it does not, by design, distinguish the underlying factors contributing to the level of functioning described (Shaffer et al. 1983), nor has it been specifically designed to capture variation in ID (Wagner 2007). Some caution in interpretation is consequently required, as CGAS scores within the clinical sample reflect overall impairment arising from multiple causes, which may include underlying cognitive impairment alongside mental health, emotional and other psychosocial factors.

As a global measure, however, the CGAS has utility in evaluating overall functional changes before and after intervention and allows direct comparisons between ID and non‐ID samples, which are not possible with disability‐specific alternative measures (Wagner et al. 2007). The naturalistic approach to the development of the CGAS (Shaffer et al. 1983) and indications of its use elsewhere clinically (National CAMHS Support Service 2010), further support its relevance to the broad CAMHS population, including those with ID. The present study, despite possible sensitivity limitations for the ID population, showed clear evidence of positive functional changes in children with ID, particularly in those with milder ID, with no indication of deterioration or harm after intervention.

There are some limitations related to sample size and representativeness. The CRIS patient data were found to be more socioeconomically deprived than the average boroughs in England, which may affect the generalisability of the findings. In addition, because of the small number of young people diagnosed with severe forms of ID and a specific comorbidity, we grouped severe and profound ID into one category and focused on the three most prevalent comorbidities. Given the heterogeneity observed within the ID sample, future research would benefit from replicating this study in other regions of England, exploring variations in intervention outcomes between severe and profound ID, and examining the effects of other comorbidities, such as anxiety and depression. Improved data linkage and sharing across electronic health records could facilitate this by enabling access to larger, more representative samples.

It is unclear what forms of intervention participants received, as treatment types are currently not available in the structured fields of the dataset, and some young people were seen by CAMHS for neurodevelopmental diagnostic assessment only rather than treatment. Further work to extract treatment types (psychosocial and pharmacological) from free‐text clinical notes is underway.

### 4.5 Clinical Implications and Recommendations

The present study found that 16.5% of the variance in CGAS improvement was attributed to service team clustering. This suggests that differences between teams may significantly influence outcomes, raising concerns about whether generic or community services can adequately support young people with ID. Where local teams lack training and resources to manage the complex presentations of ID, this may lead to unmet needs, poor outcomes, and delays in accessing specialist support. Such service gaps may perpetuate health inequalities faced by this population. Future research comparing intervention outcomes across different CAMHS teams would help inform more equitable and effective service provision.

In parallel, we identified a three‐way interaction between ID diagnosis, baseline functioning, and comorbidities, which remained significant after accounting for service team clustering. This suggests that, beyond service‐level variability, individual clinical profiles may also play a role in shaping outcomes.

The method of measuring outcomes for young people with ID warrants further consideration. The present study used the standard clinical cutoff point of 60 for CGAS in young people with and without ID. However, it remains unclear whether this threshold should be lower for those with ID, or whether alternative measures, such as the DD‐CGAS, which have more robust psychometric properties for detecting subtle changes in psychosocial functioning among this population, should be adopted. Moreover, many young people with ID accessing highly specialist CAMHS are also supported by social services. Their initial presentations may involve a mix of psychological, physical, and social problems. In such cases, intervention outcomes should be interpreted within the context of multiagency collaboration, with improvements potentially resulting from the combined input of mental health, education, and social care. Future research comparing outcomes for young people with ID who receive CAMHS and social care versus those without such involvement could clarify the added value of integrated service provision.

## 5 Conclusions

Young people with ID were more likely to have poorer psychosocial functioning at discharge than those without ID, and this was partly explained by their lower levels of psychosocial functioning at service entry. While 20% of young people with ID demonstrated clinically significant improvement at discharge, 80% remained in the clinical range and continued to experience significant symptoms, which is higher than the proportion for young people without ID (45%). The proportion of young people shifting into the nonclinical range was lower in those with more severe forms. However, the magnitude of improvement was greater in young people with severe/profound ID and a prevalent comorbidity than in those without comorbidity.

The present study confirms that young people with ID can benefit from mental health services, yet considerable progress is needed to achieve optimal outcomes. Given the important differences observed among young people with varying levels of ID severity and comorbidities, and that they received interventions from different CAMHS teams, future research should focus on exploring treatment trajectories in these subgroups and refining interventions to address their complex needs more effectively.

## Funding

This study is jointly funded by the NIHR Health and Social Care Delivery Research Programme [Project reference NIHR134922] and What Works for Early Intervention and Children's Social Care. It is also supported by the National Institute for Health and Care Research (NIHR) Applied Research Collaboration East of England (NIHR ARC EoE) at Cambridgeshire and Peterborough NHS Foundation Trust. The views expressed are those of the authors and not necessarily those of the NIHR, the Department of Health and Social Care, or the Department of Education.

## Ethics Statement

Clinical Record Interactive Search (CRIS) was approved as a dataset for secondary analysis by the Oxford C Research Ethics Committee (23/SC/0257). All data used is deidentified. Approval to conduct the study was granted in December 2022 by the CRIS Oversight Committee (22‐085).

## Conflicts of Interest

The authors declare no conflicts of interest.

## Supporting information


**Table S1:** Comparisons of missing data in Ethnicity across predictors. ‘Age when referral accepted,’ ‘age at earliest diagnosis,’ ‘first CGAS score,’ ‘IMD at index date,’ and ‘ID diagnosis’ were predictors of missingness in Ethnicity.
**Table S2:** Results of covariates covariate‐dependent missingness for the Ethnicity variable.
**Figure S1:** Diagnostic plots of multiple imputation. (a) The random patterns of the trace plots indicate good convergence. (b) The density plots of the IMD and duration of service engagement variables show good convergence between observed and imputed data. (c) The strip plot also shows good convergence between observed and imputed data for the Ethnicity variable.
**Table S3:** Sociodemographic characteristics of case and control groups before and after matching.
**Figure S2:** Love plot of mean differences before and after propensity score matching. All adjusted points (after matching) fall within the two dashed vertical lines, indicating that the balance is achieved. Standardised mean differences are displayed for continuous variables (indicated by stars).
**Table S4:** Mediation analysis for the case and control groups (Model 1). Missing data were handled using multiple imputation and compared to complete case analysis.
**Table S5:** Moderation analyses of case and control groups (Model 2). Missing data were handled using multiple imputation and compared to complete case analysis.
**Table S6:** Moderation analysis for patients with different levels of ID severity (Model 3). Missing data were handled using multiple imputation and compared to complete case analysis.
**Table S7:** CGAS scores at admission and discharge in young patients with different levels of ID severity.

## Data Availability

Data may be obtained from a third party and is not publicly available. Approval for data access can only be provided by the CRIS Oversight Committee at SLaM.
