# Supplementary material for: Young People With and Without Intellectual Disability Accessing Mental Health Services: Evaluating Psychosocial Functioning Outcomes Using Electronic Health Records
Source: J Intellect Disabil Res. 2025 Nov 29;70(2):194–205. doi: 10.1111/jir.70066 (PMC12757200; doi:10.1111/jir.70066)
Supplement: Supplementary file 1 — Table S1: Comparisons of missing data in Ethnicity across predictors. ‘Age when referral accepted,’ ‘age at earliest diagnosis,’ ‘first CGAS score,’ ‘IMD at index date,’ and ‘ID diagnosis’ were predictors of missingness in Ethnicity. Table S2: Results of covariates covariate‐dependent missingness for the Ethnicity variable. Figure S1: Diagnostic plots of multiple imputation. (a) The random patterns of the trace plots indicate good convergence. (b) The density plots of the IMD and duration of service engagement variables show good convergence between observed and imputed data. (c) The strip plot also shows good convergence between observed and imputed data for the Ethnicity variable. Table S3: Sociodemographic characteristics of case and control groups before and after matching. Figure S2: Love plot of mean differences before and after propensity score matching. All adjusted points (after matching) fall within the two dashed vertical lines, indicating that the balance is achieved. Standardised mean differences are displayed for continuous variables (indicated by stars). Table S4: Mediation analysis for the case and control groups (Model 1). Missing data were handled using multiple imputation and compared to complete case analysis. Table S5: Moderation analyses of case and control groups (Model 2). Missing data were handled using multiple imputation and compared to complete case analysis. Table S6: Moderation analysis for patients with different levels of ID severity (Model 3). Missing data were handled using multiple imputation and compared to complete case analysis. Table S7: CGAS scores at admission and discharge in young patients with different levels of ID severity. [file JIR-70-194-s001.docx]

**Table S1**. Comparisons of missing data in Ethnicity across predictors. ‘Age when referral accepted’, ‘age at earliest diagnosis’, ‘First CGAS score’, ‘IMD at index date’, and ‘ID diagnosis’ were predictors of missingness in Ethnicity.

| **Missing data analysis: Ethnicity** | | **Not missing** | **Missing** | ***p* value** |
| --- | --- | --- | --- | --- |
| Age when referral accepted | Mean (SD) | 12.1 (3.7) | 12.5 (3.6) | < 0.001 |
| Age at earliest diagnosis | Mean (SD) | 12.0 (3.6) | 12.8 (3.5) | < 0.001 |
| First CGAS score | Mean (SD) | 51.7 (12.4) | 53.2 (12.4) | < 0.001 |
| Last CGAS score | Mean (SD) | 61.7 (14.0) | 61.4 (14.2) | 0.54 |
| Gender | Female | 10171 (94.4) | 603 (5.6) | 0.52 |
|  | Male | 10427 (94.1) | 653 (5.9) |  |
|  | Not Specified | 12 (100.0) | 0 (0.0) |  |
|  | Other | 81 (96.4) | 3 (3.6) |  |
|  | (Missing) | 1 (50.0) | 1 (50.0) |  |
| IMD at index date | Mean (SD) | 27.9 (11.9) | 25.4 (11.7) | < 0.001 |
| Duration of service engagement | Mean (SD) | 70.2 (88.7) | 34.5 (37.3) | < 0.001 |
| ID diagnosis | No (0) | 18798 (94.2) | 1168 (5.8) | 0.03 |
|  | Yes (1) | 1894 (95.4) | 92 (4.6) |  |

**Table S2.** Results of covariates covariate-dependent missingness for the Ethnicity variable

|  | **Estimate** | **Standard Error** | ***p* value** |
| --- | --- | --- | --- |
| (Intercept) | -1.13 | 0.10 | < 0.001 |
| Age when referral accepted | -0.02 | 0.01 | 0.03 |
| Age at earliest diagnosis | -0.03 | 0.01 | < 0.001 |
| First CGAS score | 0.01 | 0.00 | < 0.001 |
| IMD at index date | 0.04 | 0.00 | < 0.001 |
| Duration of service engagement | -0.00 | 0.00 | 0.03 |
| ID diagnosis | 0.28 | 0.05 | < 0.001 |

**Figure S1**. Diagnostic plots of multiple imputation. (a) The random patterns of the trace plots indicate good convergence. (b). The density plots of the IMD and duration of service engagement variables show good convergence between observed and imputed data.(c) The strip plot also shows good convergence between observed and imputed data for the Ethnicity variable.


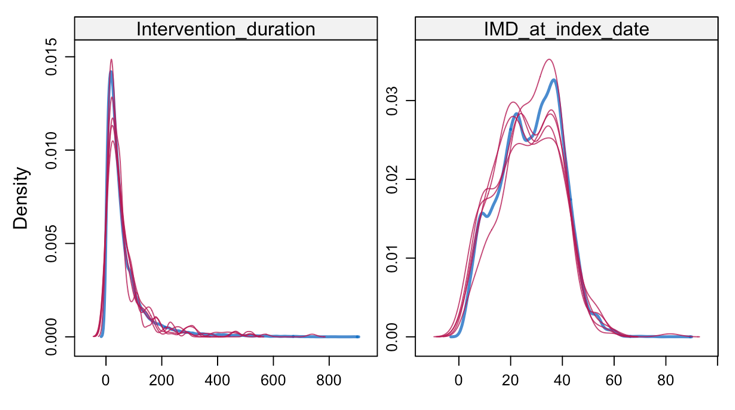

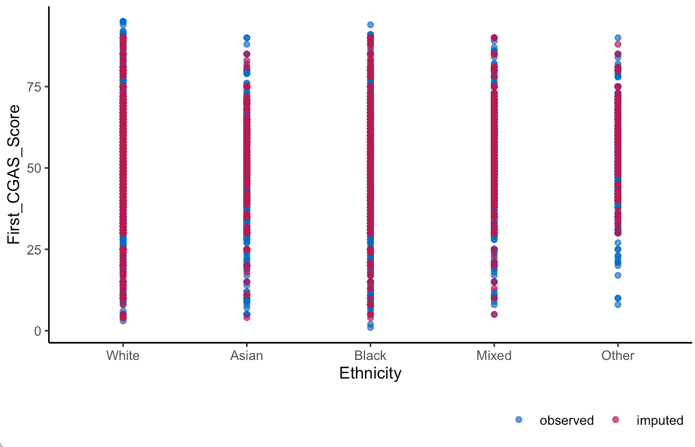


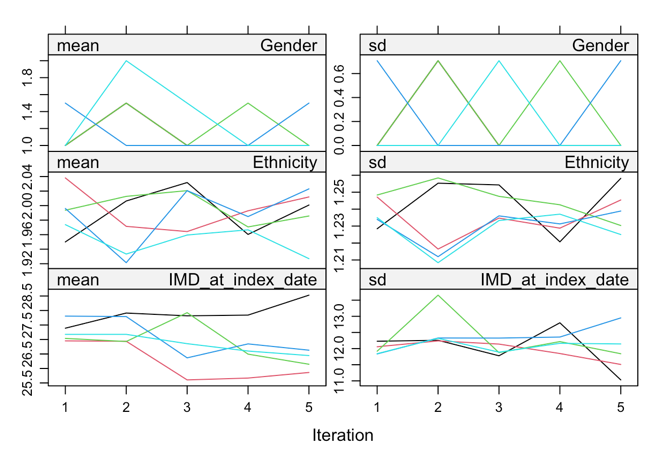

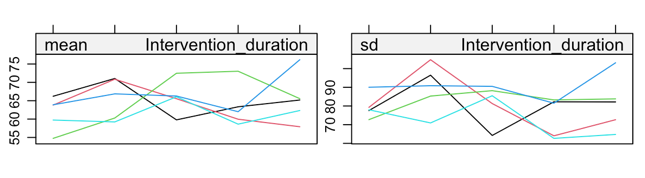


**a**

**b**

**c**

**Table S3.** Sociodemographic characteristics of case and control groups before and after matching.

|  |  | **Before matching** | | | **After matching** | | |
| --- | --- | --- | --- | --- | --- | --- | --- |
|  |  | **Case** | **Control** | **Mean difference** | **Case** | **Control** | **Mean difference** |
| Age when referral accepted* | mean | 11.17 | 12.19 | -0.272 | 11.18 | 11.09 | 0.016 |
| Ethnicity White | *N*(%) | 941 (47.4) | 10452 (55.8) | -0.124 | 991 (49.9) | 1941 (49.0) | 0.010 |
| Ethnicity Asian | *N*(%) | 142 (7.2) | 868 (4.6) | 0.108 | 149 (7.5) | 309 (7.8) | -0.004 |
| Ethnicity Black | *N*(%) | 602 (30.3) | 4777 (25.2) | 0.139 | 627 (31.6) | 1268 (32.0) | -0.005 |
| Ethnicity Mixed | *N*(%) | 143 (7.2) | 2040 (10.8) | -0.118 | 150 (7.6) | 315 (7.9) | -0.004 |
| Ethnicity Other | *N*(%) | 66 (3.3) | 661 (3.6) | 0.001 | 69 (3.5) | 131 (3.3) | 0.002 |
| Gender Female | *N*(%) | 628 (31.6) | 9722 (50.8) | -0.413 | 628 (31.6) | 1227 (31.0) | 0.007 |
| Gender Male | *N*(%) | 1358 (68.4) | 10146 (48.7) | 0.423 | 1358 (68.4) | 2737 (69.0) | -0.007 |
| Gender Not Specified | *N*(%) | 0 (0) | 12 (0.1) | -0.026 | 0 (0) | 0 (0) | 0.000 |
| Gender Other | *N*(%) | 0 (0) | 84 (0.4) | -0.068 | 0 (0) | 0 (0) | 0.000 |
| Duration of service engagement | mean | 97.04 | 65.27 | 0.26 | 97.21 | 94.19 | 0.030 |

* Standardised mean differences are displayed for continuous variables. The rest of the variables are displayed as raw mean differences.


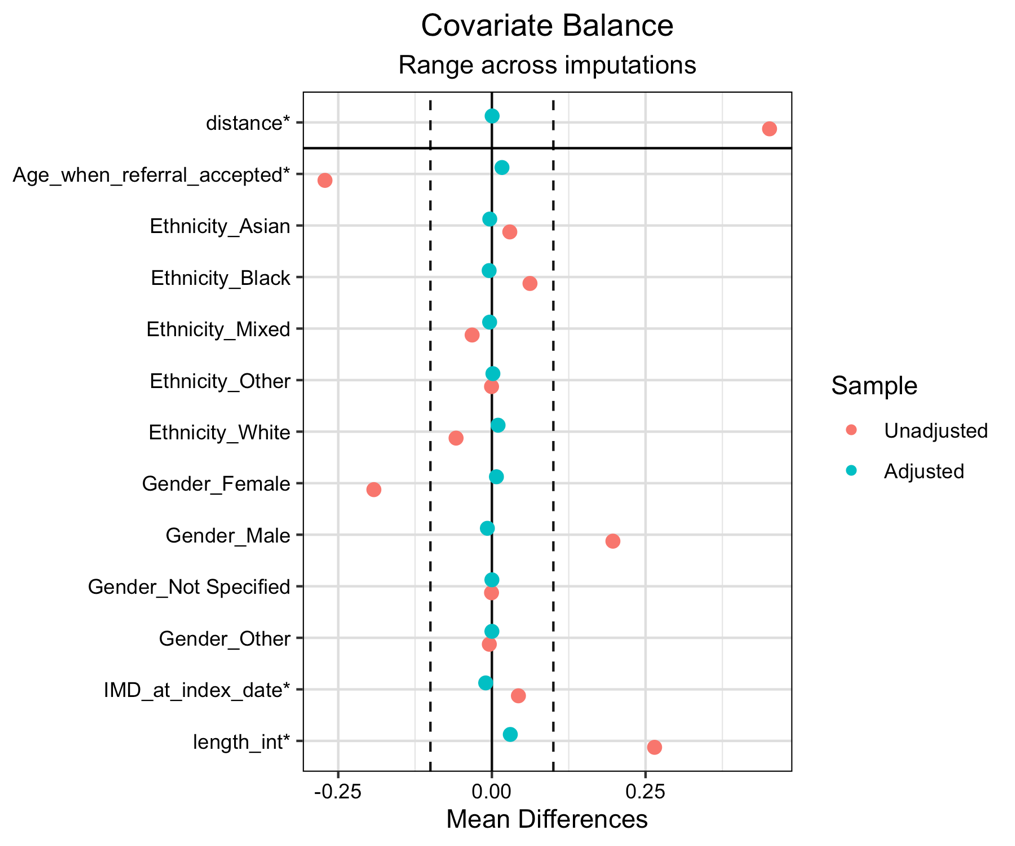


**Figure S2.** Love plot of mean differences before and after propensity score matching. All adjusted points (after matching) fall within the two dashed vertical lines, indicating that the balance is achieved. Standardised mean differences are displayed for continuous variables (indicated by stars).

**Table S4**. Mediation analysis for the case and control groups (Model 1). Missing data were handled using multiple imputation and compared to complete case analysis.

|  | **Multiple imputation ^1^** | | **Complete case analysis** | |
| --- | --- | --- | --- | --- |
|  | *β* | 95% CI | *β* | 95% CI |
| Direct effect ^2^ | -5.30 | [-6.50; -4.05] | -7.03 | [-7.71; -6.34] |
| Total indirect effect ^3^ | -6.31 | [-7.44; -5.22] | -7.56 | [-8.13; -7.02] |
| Total effect ^4^ | -11.61 | [-13.04; -10.15] | -14.60 | [-15.33; -13.76] |

^1^ Currently, no packages are available in R for testing the mediation effects of multiple imputed datasets

^2^ ID case -> Last CGAS Score (adjusting for first CGAS score)

^3^ ID case -> First CGAS Score -> Last CGAS Score

^4^ ID case -> Last CGAS Score

**Table S5.** Moderation analyses of case and control groups (Model 2). Missing data were handled using multiple imputation and compared to complete case analysis.

| **Dependent variable: Last CGAS score** | | | | | | | | | | | |
| --- | --- | --- | --- | --- | --- | --- | --- | --- | --- | --- | --- |
|  | **Multiple imputation** | | | | | | | **Complete case analysis** | | | |
|  | *β* | | | Standard error | | | 95% CI | *β* | Standard error | | 95% CI |
| First CGAS Score | 0.68 | | | 0.01 | | | [0.66; 0.70]*** | 0.66 | 0.01 | | [0.64; 0.68]*** |
| ID | -5.17 | | | 0.36 | | | [-5.87; -4.47]*** | -7.04 | 0.34 | | [-7.72; -6.37]*** |
| Age when referral accepted | 0.25 | | | 0.04 | | | [0.14; 0.31]*** | 0.20 | 0.04 | | [0.12; 0.28]*** |
| Sex (Male) | -1.19 | | | 0.34 | | | [-1.85; -0.53]*** | -2.04 | 0.33 | | [-2.68; -1.39]*** |
| Ethnicity |  | | |  | | |  |  |  | |  |
| Asian | 0.98 | | | 0.57 | | | [-0.15; 2.10] | 0.73 | 0.57 | | [-0.38; 1.84] |
| Black | -0.52 | | | 0.36 | | | [-1.23; 0.19] | 0.06 | 0.35 | | [-0.63; 0.75] |
| Mixed | 0.01 | | | 0.56 | | | [-1.09; 1.11] | 0.20 | 0.57 | | [-0.92; 1.31] |
| Other | 0.65 | | | 0.92 | | | [-1.16; 2.46] | 1.11 | 0.88 | | [-0.62; 2.84] |
| IMD | -0.03 | | | 0.01 | | | [-0.05 0.00] | -0.007 | 0.01 | | [-0.03; 0.02] |
| Duration of service engagement | 0.007 | | | 0.00 | | | [0.004; 0.009]*** | 0.005 | 0.00 | | [0.002; 0.008]*** |
| **Step 1 : First CGAS x ID** | | | | | | | | | | | |
| First CGAS Score | | 0.56 | | | 0.04 | [0.52; 0.59]*** | | 0.53 | 0.02 | [0.50; 0.55]*** | |
| ID case (0;1) | | -16.66 | | | 2.23 | [-19.05; -14.27]*** | | -21.20 | 1.11 | [-23.37; -19.04]*** | |
| First CGAS x ID | | 0.24 | | | 0.04 | [0.19; 0.29]*** | | 0.30 | 0.02 | [0.26; 0.35]*** | |
| **Step 2 : First CGAS x Prevalent Comorbidity** | | | | | | | | | | | |
| **(1) PDD model** | | | | | | | | | | | |
| First CGAS Score | 0.65 | | | | 0.02 | [0.62; 0.68]*** | | 0.64 | 0.01 | [0.61; 0.66]*** | |
| PDD (0/1) | -10.14 | | | | 1.18 | [-12.45; -7.83]*** | | -13.84 | 1.11 | [-16.02; -11.65]*** | |
| First CGAS x PDD | 0.13 | | | | 0.02 | [0.08; 0.18]*** | | 0.17 | 0.02 | [0.13; 0.22]*** | |
| **(2) HD model** | | | | | | | | | | | |
| First CGAS Score | | 0.73 | | | 0.01 | [0.71; 0.76]*** | | 0.76 | 0.01 | [0.74; 0.79]*** | |
| HD (0/1) | | 0.07 | | | 1.53 | [-2.93; 3.07] | | -0.36 | 1.51 | [-3.32; 2.59] | |
| First CGAS x HD | | -0.03 | | | 0.03 | [-0.09; 0.03] | | -0.04 | 0.03 | [-0.10; 0.02] | |
| **(3) CD model** | | | | | | | | | | | |
| First CGAS Score | | 0.74 | | | 0.01 | [0.71; 0.76]*** | | 0.77 | 0.01 | [0.74; 0.79]*** | |
| CD (0;1) | | 4.80 | | | 2.33 | [0.22; 9.38]* | | 3.89 | 2.25 | [-0.53; 8.31] | |
| First CGAS x CD | | -0.12 | | | 0.05 | [-0.21; -0.03]** | | -0.11 | 0.05 | [-0.21; -0.03]** | |
| **Step 3 : Three-way interaction analyses** | | | | | | | | | | | |
| **(1) PDD model** | | | | | | | | | | | |
| First CGAS Score | | | 0.51 | 0.02 | | [0.47; 0.55]*** | | 0.48 | 0.02 | [0.44; 0.50]*** | |
| ID case (0;1) | | | -19.59 | 1.62 | | [-22.77; -16.42]*** | | -23.08 | 1.51 | [-26.04; -20.12]*** | |
| PDD (0;1) | | | -14.68 | 2.34 | | [-19.27; -10.08]*** | | -14.79 | 2.12 | [-18.95; -10.64]*** | |
| First CGAS x ID | | | 0.29 | 0.03 | | [0.23; 0.35]*** | | 0.34 | 0.03 | [0.28; 0.40]*** | |
| First CGAS x PDD | | | 0.20 | 0.04 | | [0.12; 0.29]*** | | 0.19 | 0.04 | [0.11; 0.27]*** | |
| ID x PDD | | | 13.79 | 2.77 | | [8.35; 19.23]*** | | 13.32 | 2.61 | [8.20; 18.44]*** | |
| First CGAS x ID x PDD | | | -0.22 | 0.06 | | [-0.33; -0.11]*** | | -0.21 | 0.05 | [-0.31; -0.10]*** | |
| **(2) HD model** | | | | | | | | | | | |
| First CGAS Score | | 0.55 | | | 0.02 | [0.51; 0.58]*** | | 0.51 | 0.02 | [0.48; 0.54]*** | |
| ID case (0;1) | | -18.81 | | | 1.31 | [-21.38; -16.24]*** | | -23.72 | 1.19 | [-26.05; -21.38]*** | |
| HD (0;1) | | -7.75 | | | 2.70 | [-13.06; -2.44]** | | -10.75 | 2.56 | [-15.76; -5.74]*** | |
| First CGAS x ID | | 0.27 | | | 0.03 | [0.22; 0.32]*** | | 0.34 | 0.02 | [0.29; 0.39]*** | |
| First CGAS x HD | | 0.10 | | | 0.05 | [0.00; 0.20]** | | 0.13 | 0.05 | [0.04; 0.22]** | |
| ID x HD | | 13.11 | | | 3.28 | [6.69; 19.54]*** | | 18.06 | 3.19 | [11.79; 24.32]*** | |
| First CGAS x ID x HD | | -0.20 | | | 0.07 | [-0.32; -0.07]** | | -0.27 | 0.06 | [-0.40; -0.14]*** | |
| **(3) CD model** | | | | | | | | | | | |
| First CGAS Score | | 0.56 | | | 0.02 | [0.52; 0.59]*** | | 0.52 | 0.02 | [0.49; 0.55]*** | |
| ID case (0;1) | | -17.48 | | | 1.24 | [-19.91; -15.05]*** | | -22.16 | 1.14 | [-24.39; -19.93]*** | |
| CD (0;1) | | -2.59 | | | 3.64 | [-9.81; 4.62] | | -5.35 | 3.29 | [-11.80; 1.11] | |
| First CGAS x ID | | 0.25 | | | 0.02 | [0.20; 0.29]*** | | 0.31 | 0.02 | [0.27; 0.36]*** | |
| First CGAS x CD | | -0.01 | | | 0.07 | [-0.15; 0.12] | | 0.03 | 0.06 | [-0.10; 0.14] | |
| ID x Conduct disorder | | 7.78 | | | 4.75 | [-1.57; 17.13] | | 12.32 | 4.57 | [3.35; 21.29]** | |
| First CGAS x ID x CD | | -0.05 | | | 0.10 | [-0.24; 0.15] | | -0.14 | 0.10 | [-0.33; 0.05] | |

Note:

(1) **p* < 0.05, ***p* < 0.01, ****p* < 0.001

(2) Reference group: ID/ PDD/ HD/ CD = 0 (No diagnosis)

(3) PDD = pervasive developmental disorders; HD = hyperkinetic disorders; CD = conduct disorders

(4) All models are adjusted for age when referral accepted, sex, ethnicity, and duration of service engagement. A random intercept for CAMHS service team was included to account for clustering

**Table S6**. Moderation analysis for patients with different levels of ID severity (Model 3). Missing data were handled using multiple imputation and compared to complete case analysis.

| **Dependent variable:** Δ **CGAS improvement** | | | | | | | | | | | | | | | |  |
| --- | --- | --- | --- | --- | --- | --- | --- | --- | --- | --- | --- | --- | --- | --- | --- | --- |
|  |  | | | **Multiple Imputation** | | | | |  | | | **Complete Case Analysis** | | | |  |
|  | *β* | | | | Standard error | | | 95% CI | *β* | | Standard error | | | | 95% CI |  |
| **Model 1** | | | | | | | | | | | | | | | |  |
| Moderate ID | | | -1.09 | | | 0.58 | [-2.22; 0.05] | | | -1.52 | 0.60 | | [-2.69; -0.34]* | | | |
| Severe ID | | | -0.52 | | | 0.68 | [-1.84; 0.81] | | | -0.83 | 0.68 | | [-2.16; 0.50] | | | |
| Profound ID | | | -1.34 | | | 1.47 | [-4.22; 1.55] | | | -2.66 | 1.56 | | [-5.72; 0.39] | | | |
| Other/unspecified ID | | | 2.11 | | | 0.85 | [0.45; 3.77]* | | | 2.07 | 0.90 | | [0.31; 3.83]* | | | |
| **Model 2: Two-way interaction analyses** | | | | | | | | | | | | | | | |  |
| **(1) PDD model** | | | | | | | | | | | | | | | |  |
| Moderate ID | | | -1.93 | | 0.80 | | [-3.49; -0.36]* | | | -1.79 | 0.87 | | | [-3.49; -0.09]* | |  |
| Severe/Profound ID | | | -7.59 | | 1.12 | | [-9.78; -5.39]*** | | | -2.59 | 1.13 | | | [-4.80; -0.36]* | |  |
| Other/Unspecified ID | | | 1.31 | | 1.17 | | [-0.10; 3.61] | | | 0.70 | 1.27 | | | [-1.79; 3.18] | |  |
| PDD (0; 1) ^b^ | | | -2.00 | | 0.74 | | [-3.45; -0.55]** | | | -1.79 | 0.79 | | | [-3.34; -0.24]* | |  |
| Moderate ID x PDD | | | -0.40 | | 1.12 | | [-2.60; 1.80] | | | 1.12 | 1.22 | | | [-1.28; 3.51] | |  |
| Severe/Profound ID x PDD | | | 3.42 | | 1.30 | | [0.86; 5.97]** | | | 2.95 | 1.42 | | | [0.16; 5.74]* | |  |
| Other/Unspecified ID x PDD | | | 0.06 | | 1.67 | | [-3.22; 3.33] | | | 3.10 | 1.80 | | | [-0.42; 6.62] | |  |
| **(2) HD model** | | | | | | | | | | | | | | | |  |
| Moderate ID | | -2.94 | | | | 0.65 | [-4.22; -1.67]*** | | | -2.14 | 0.69 | | | [-3.49; -0.80]** | |  |
| Severe/Profound ID | | -6.14 | | | | 0.79 | [-7.70; -4.59]*** | | | -1.97 | 0.74 | | | [-3.43; -0.51]** | |  |
| Other/Unspecified ID | | 1.20 | | | | 0.97 | [-0.70; 3.11] | | | 1.65 | 1.05 | | | [-0.40; 3.71] | |  |
| HD (0; 1) | | -1.14 | | | | 0.83 | [-2.78; 0.50] | | | -1.27 | 0.89 | | | [-3.02; 0.48] | |  |
| Moderate ID x HD | | 2.08 | | | | 1.31 | [-0.49; 4.65] | | | 2.57 | 1.42 | | | [-0.21; 5.35] | |  |
| Severe/Profound ID x HD | | 3.50 | | | | 1.46 | [0.63; 6.36]* | | | 4.08 | 1.55 | | | [1.03; 7.13]** | |  |
| Other/Unspecified ID x HD | | -0.08 | | | | 1.87 | [-3.76; 3.58] | | | 1.46 | 2.01 | | | [-2.48; 5.41] | |  |
| **(3) CD model** | | | | | | | | | | | | | | | |  |
| Moderate ID | | -2.58 | | | | 0.59 | [-3.75; -1.42]*** | | | -1.71 | 0.62 | | | [-2.93; -0.49]** | |  |
| Severe/Profound ID | | -5.54 | | | | 0.75 | [-7.01; -4.07]*** | | | -1.30 | 0.68 | | | [-2.63; 0.02] | |  |
| Other/Unspecified ID | | 0.83 | | | | 0.87 | [-0.88; 2.53] | | | 1.63 | 0.93 | | | [-0.20; 3.45] | |  |
| CD (0; 1) | | 0.60 | | | | 1.30 | [-1.94; 3.14] | | | 0.40 | 1.39 | | | [-2.31; 3.12] | |  |
| Moderate ID x CD | | 2.28 | | | | 2.12 | [-1.89; 6.44] | | | 3.17 | 2.32 | | | [-1.39; 7.73] | |  |
| Severe/Profound ID x CD | | 2.06 | | | | 2.16 | [-2.17; 6.29] | | | 3.07 | 2.31 | | | [-1.46; 7.60] | |  |
| Other/Unspecified ID x CD | | 4.70 | | | | 3.04 | [-1.27; 10.67] | | | 5.79 | 3.34 | | | [-0.76; 12.34] | |  |

Note:

(1) **p* < 0.05, ***p* < 0.01, ****p* < 0.001

(2) ICD-10 coding system: F70 = Mild ID, F71 = Moderate ID, F72 = Severe ID, F73 = Profound ID, F78 = Other ID, F79 = Unspecified ID. ‘F70 Mild ID’ is the reference group for ID severity.

(3) Similar to the case-control study, ‘Age when referral was accepted’, ‘sex’, and ‘ethnicity’ were adjusted for in the regression models.

(4) PDD = pervasive developmental disorders; HD = hyperkinetic disorders; CD = conduct disorders. Reference group: PDD/ HD/ CD = 0 (No diagnosis)

(5) All models are adjusted for age when referral accepted, sex, ethnicity, and duration of service engagement. A random intercept for CAMHS service team was included to account for clustering.

**Table S7.** CGAS scores at admission and discharge in young patients with different levels of ID severity.

| **Measure** |  | **All ID** | **Mild ID** | **Moderate ID** | **Severe/**  **profound ID** | **Other/**  **unspecified ID** |
| --- | --- | --- | --- | --- | --- | --- |
| Observations | episode of care | 1986 | 800 | 558 | 447 | 181 |
| First CGAS Score | mean (SD) | 41.5 (14.6) | 47.7 (11.6) | 42.6 (12.6) | 28.8 (14.0) | 41.7 (12.9) |
| Last CGAS Score | mean (SD) | 47.9 (15.9) | 54.6 (12.2) | 48.2 (13.9) | 34.7 (16.3) | 50.2 (14.1) |
| CGAS Changes | mean (SD) | 6.48 (10.6) | 6.9 (10.9) | 5.63 (10.3) | 5.89 (10.0) | 8.48 (11.7) |
| First CGAS < 61 | *N* (%) | 1837 (92.5) | 711 (88.9) | 518 (92.8) | 441 (98.7) | 167 (92.3) |
| Last CGAS < 61 | *N* (%) | 1587 (80.0) | 566 (70.8) | 468 (83.9) | 417 (93.3) | 136 (75.1) |
| **PDD** |  |  |  |  |  |  |
| Observations | episode of care | 1064 | 304 | 333 | 331 | 96 |
| First CGAS Score | mean (SD) | 37.8 (14.1) | 46.0 (12.0) | 39.6 (11.8) | 28.4 (13.2) | 38.1 (12.0) |
| Last CGAS Score | mean (SD) | 44.0 (15.2) | 51.7 (12.0) | 44.9 (13.4) | 35.0 (15.4) | 47.5 (13.0) |
| CGAS Changes | mean (SD) | 6.18 (10.5) | 5.74 (10.6) | 5.28 (9.80) | 6.55 (10.3) | 9.42 (12.2) |
| First CGAS < 61 | *N* (%) | 1018 (95.7) | 274 (90.1) | 322 (96.7) | 329 (99.4) | 93 (96.9) |
| Last CGAS < 61 | *N* (%) | 927 (87.1) | 237 (78.0) | 297 (89.2) | 312 (94.3) | 81 (84.4) |
| **HD** |  |  |  |  |  |  |
| Observations | episode of care | 456 | 209 | 113 | 86 | 48 |
| First CGAS Score | mean (SD) | 41.9 (12.7) | 47.3 (10.1) | 40.7 (12.0) | 30.8 (12.4) | 40.7 (11.7) |
| Last CGAS Score | mean (SD) | 49.5 (14.4) | 53.8 (11.9) | 48.2 (14.6) | 40.1 (14.9) | 50.2 (14.7) |
| CGAS Changes | mean (SD) | 7.59 (11.8) | 6.51 (11.0) | 7.43 (13.0) | 9.33 (12.1) | 9.52 (11.0) |
| First CGAS < 61 | *N* (%) | 432 (94.7) | 193 (92.3) | 107 (94.7) | 86 (100) | 46 (95.8) |
| Last CGAS < 61 | *N* (%) | 369 (80.9) | 157 (75.1) | 97 (85.8) | 78 (90.7) | 37 (77.1) |
| **CD** |  |  |  |  |  |  |
| Observations | episode of care | 155 | 68 | 34 | 37 | 16 |
| First CGAS Score | mean (SD) | 40.9 (12.1) | 45.9 (9.8) | 39.0 (13.3) | 33.9 (11.1) | 40.1 (11.5) |
| Last CGAS Score | mean (SD) | 49.7 (14.6) | 53.1 (12.6) | 48.2 (17.4) | 43.5 (14.9) | 52.4 (10.5) |
| CGAS Changes | mean (SD) | 8.75 (12.1) | 7.26 (8.76) | 9.15 (16.8) | 9.57 (11.7) | 12.3 (13.9) |
| First CGAS < 61 | *N* (%) | 149 (96.1) | 63 (92.6) | 33 (97.1) | 37 (100) | 16 (100) |
| Last CGAS < 61 | *N* (%) | 126 (81.3) | 54 (79.4) | 28 (82.4) | 32 (86.5) | 12 (75.0) |

Note:

(1) ICD-10 coding system: F70 = Mild ID, F71 = Moderate ID, F72 = Severe ID, F73 = Profound ID, F78 = Other ID, F79 = Unspecified ID

(2) PDD = pervasive developmental disorders; HD = hyperkinetic disorders; CD = conduct disorders.
